# Quantitative Quality Evaluation of Software Products by Considering Summary and Comments Entropy of a Reported Bug

**DOI:** 10.3390/e21010091

**Published:** 2019-01-19

**Authors:** Madhu Kumari, Ananya Misra, Sanjay Misra, Luis Fernandez Sanz, Robertas Damasevicius, V.B. Singh

**Affiliations:** 1 Department of Computer Science, Delhi College of Arts and Commerce, Delhi 110023, India; 2 Department of Informatics, Technical University of Munich, 80333 Munich, Germany; 3 Department of Computer Engineering, Atilim University, Ankara 06830, Turkey; 4 Department of Electrical and Information Engineering, Covenant University, Ota, Ogun State 112212, Nigeria; 5 Department of Computer Science, University of Alcala, 28801 Madrid, Spain; 6 Department of Software Engineering, Kaunas University of Technology, 44249 Kaunas, Lithuania

**Keywords:** entropy, software reliability growth model, non-homogenous poissonprocess, bug summary, bug comments, veracity, big data, bug dependency

## Abstract

A software bug is characterized by its attributes. Various prediction models have been developed using these attributes to enhance the quality of software products. The reporting of bugs leads to high irregular patterns. The repository size is also increasing with enormous rate, resulting in uncertainty and irregularities. These uncertainty and irregularities are termed as veracity in the context of big data. In order to quantify these irregular and uncertain patterns, the authors have appliedentropy-based measures of the terms reported in the summary and the comments submitted by the users. Both uncertainties and irregular patterns have been taken care of byentropy-based measures. In this paper, the authors considered that the bug fixing process does not only depend upon the calendar time, testing effort and testing coverage, but it also depends on the bug summary description and comments. The paper proposed bug dependency-based mathematical models by considering the summary description of bugs and comments submitted by users in terms of the entropy-based measures. The models were validated on different Eclipse project products. The models proposed in the literature have different types of growth curves. The models mainly follow exponential, S-shaped or mixtures of both types of curves. In this paper, the proposed models were compared with the modelsfollowingexponential, S-shaped and mixtures of both types of curves.

## 1. Introduction

Software is indispensable in modern society. The development of software follows different process models. During the last few decades, due to the exponential growth in software applications, pressure has been mounting on the software industries to produce reliable software in a short space of time. Consequently, software development life cycle models have shifted from the traditional waterfall model to extreme programming and object-oriented life cycle models. In each life phase of software development, programmers try to minimizeinherent errors by walk-through and code inspectionmethods.Recently, a paradigm shift has been taking place in software development due to advancements in the communication technology which has resulted to the emergence of open source software. The evolution of software takes place through an active participation of different users and developers working from different geographical locations. Among the various issues raised by users around the world one may highlight bugs, new feature introduction, and feature improvement that needs to be incorporated in the software over a long run. Once the issues raisedare reported on the issue tracking system, they are verified, diagnosed and then fixed. The fixing of bugs follows different growth curves and it has been modeled quantitatively by using different software reliability growth models. These mathematical models quantitatively evaluate the reliability level of software in terms of the number ofbugs removed from the software.The reliability level measures the quality of software products as it is one of theimportant software quality attributes which can be measured and predicted [1]. To incorporate the issues arising from the use of these software products, the source code of software isfrequentlychanged. During the changes in the source code of the software, a set of files gets modified. These source code modifications in different files, for a given period of time, follow a specific probability distribution. This has been quantified in [2] by applying Shannon’s entropy proposed in [3]. This entropy measure has been used to quantify theuncertainty arising from source code changes. In [4], the authors proposed models to predict the complexity of code changes, i.e., the entropy that can be diffused into the software overa period of time. The authors also proposed to develop entropy-based models to predict the bugs that can occurin software due to code changes [5]. The entropy has also been used to predict the software code smell [6]. A review of the literature in this regard, revealed that an NHPP-based SRGM hasbeen proposed to evaluate the reliability level of the software in terms of the number of bugs fixed and the number remaining. The information about the bugs remaining in the software may affect the software quality. The authors thus developed SRGM by considering the software development environment and different environmental factors. The SRGM is based on calendar time, testing effort and testing coverage [7,8,9,10,11,12,13,14,15,16,17,18,19,20]. In another study, various SRGMweredevelopedby considering the dependency of bugs. The bugs are dependent in the sense that the removal/fixing of bugs relies upon the removal of other bugs on which these bugs are dependent [8,12,13]. During the study of SRGM, it was observed that the models developed in the literature did not take into account the intrinsic characteristics of the bugs which were reported by all the users from various geographical locations. The models developed in the literature was also observed not to have addressed the diverse comments submitted by different developers in the process of bug fixing. Until the descriptions of the bugs areclearly stated, the root cause of the problems affecting smooth operations cannot be traced, hence the bugs cannot be appropriately fixed. It is thus the assumption of this paper that that the fixing of the bugs correlates highly with theappropriate identification and description of the issues associated with the bugs, as reported by the users. Across the different latitude and longitude of the globe, users and developers report bugs for an open source software to bug tracking systems. The size of software repositories which consists of source code, bugs and archive communication are increasing with enormous rates. The increasing size of software repositories and heterogeneous nature of this semi-structured data leads it to suffer from veracity issues. Veracity is an important attribute of Big Data and refers to biases, noise, uncertainty and distortions in data. In a recent study, authors have used Shannon’s entropy to learn from uncertainty for big data [21]. The reporting of bugs on bug tracking systems also leads to highly irregular patterns and it has also resulted in uncertainty. Both uncertainty and irregular patterns have been taken care by entropy-based measures, i.e.,summary_entropy metric and comment_entropy metric.

The reporting of bugs and others issues on issue tracking systems generates software repositories. These repositories help in software evolution. On the other hand, irregular patterns observed inthese repositories negatively affects the software quality. Once prediction models based on machine learning techniques and mathematical models are developed, the performance of the built in classifiers and models could be significantly degradedif care is not taken to avoid the emergenceof irregular patterns. In addition, where the irregular patterns are not measured, the built model’s accuracy will be degraded.

**Figure 1:**
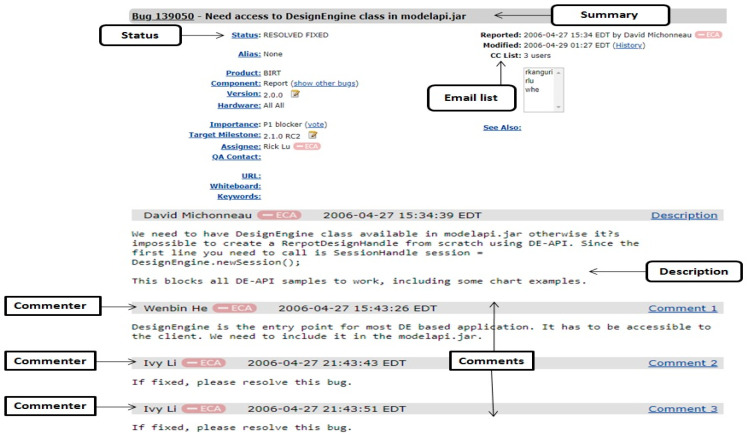
A part of the bug report for bug id 139050 of BIRT products of Eclipse projects with its three comments and summary.

The summary attribute contains the textual description of bug reports. It plays the major role in predicting severityand priority of newly incoming bug reports. The comments are attached to the bug by users. The numbers of comments filed and counted are attached to a bug report. The textual description of user comments can affect the comprehensibility of a bug report.Therefore, it assists in fixing of a bug. Number of comments, and occurrences of terms, are the indicators of the software quality. Figure 1 shows a bug report of bug id 139050 of BIRT products of the Eclipse project.

The authors of this paper have proposed models to predict the potential number of bugs to be fixed over a long run in the software. To understand the effect of summary_entropy and comment_entropy metric in the bug fixing process, the following cases have been designed:

Case 1: Prediction of latent bugs based on calendar time (month).
Case 2: Prediction of latent bugs based on summary_entropy.
Case 3: Prediction of latent bugs based on comment_entropy.

These cases are summarized as follows:
Case 1Time vs. bugsIn this case, the software reliability growth models in [7,8,9,12,14,15] have been used to predict the potential bugs lying dormant in the software.Case 2Summary_entropyvs. bugsIn this case, summary_entropy based bug prediction models have been proposed.Case 3Comment_entropyvs. bugsIn this case, comment_entropy based bug prediction models have been proposed.

The summary and comment entropy is also a function of time. Consequently, the proposed models are alsopredicting the number of bugs in a given time window.The proposed models have been empirically validated on different products of Eclipse project. The performance of the proposed modelshave been validated using different performance measures, namely *R*^2^, bias, variation, mean squared error (MSE) and root mean squared prediction error (RMSPE).

The rest of the paper is organized as follows: Section 2 discusses the data collection and model building for bug prediction approaches. Section 3 describes the result and analysis. Section 4 deals with related works and finally, the paper is concluded in Section 5 with future research directions.

## 2. Data Collection, Preprocessing and Model Building for Bug Prediction

### 2.1. Data Collection

To validate the entropy-based proposed models, the authors considered different products of the Eclipse project [22]. The reports of the bug were taken for status as “CLOSED”, “RESOLVED” and “VERIFIED” and resolution as “FIXED” and “WORKSFORME”. Table 1 shows the number of bug reports in each product of the Eclipse project.

### 2.2. Extraction ofthe Terms and Its Weight Using Summary Attributes

Summary weight attribute is derived from the bug attributessubmitted by the users. To compute the summary weight of a reported bug, the authors pre-processed the bug summary in Rapid Miner tools with the steps Tokenization, Stop Word Removal, Stemming to base stem, Feature Reduction and InfoGain [23].

### 2.3. Entropy

The primary objective of software development is to deliver high quality product at low cost.Bug reporting on software repository system is inan irregular state. Irregularity leads to uncertainty. The size of the software repositoriesis also growing at an enormous rate. This increased size usually has a lot of noise and uncertainty. The representation, measurement, modeling, and processing of uncertainty embedded throughout the data analysis process has a significant impact on the performance of learning from software repositories. If these uncertainties and noises are not handled properly, the performance of the learning strategy can be greatly reduced. To combine and consider these two phenomena, the authors utilized entropy as an attribute. Entropy is used to enhance the software project quality. In general, entropyis a measure of uncertainty in a software system. This paper thus calculated the summary_entropy and comment_entropyfor model building using Shannon’s entropy, where a random variable is defined by *A* = {*a*_1_, *a*_2_, *a*_3_,…, *a_n_*} and its probability distribution is *P* = {*p*_1_, *p*_2_, *p*_3_,…, *p_n_*}, the random uncertainty is measured by Shannon’sentropy, *Sn* is defined as:

(1) Sn=−pilog2pi

In the case of summary_entropy, *p* is calculated as:

$$p_{i}=\frac{Total\;number\;of\;occurence\;of\;terms\;in\;i^{th}\;bug\;report}{Total\;number\;of\;terms}$$

In the case of comment_entropy, *p* is calculated as:

$$p_{i}=\frac{Number\;of\;comments\;in\;i^{th}\;bug\;report}{Total\;number\;of\;comments}$$

In this study, the authors considered top 200 terms based on weight from the corpus of total terms. For each bug report, efforts was made to count the summary terms found in the set of 200 top terms and after which it was divided by the total number of terms considered in the study, i.e., 200.

### 2.4. Software Reliability Growth Modeling

The software reliability growth models measure the reliability growth of software in terms of number of bugs fixed in respect of the execution of the program. The software reliability growth models available in literature follow different types of growth curves. The growth curve may be exponential, S-shaped or some mix of both. There is also a separate category of models which incorporate the concept of bug dependency and followan S-shaped growth curve. The models developed in the literature are based on either calendar time or testing effort functions. In this paper, the auhors developed software reliability growth models based on summary and comment entropy. To validate the performance of the proposed models, the paper comparesthe software reliability growth models which follow exponential, S-shaped and or a mixture of both growth curves.

**Figure 2:**
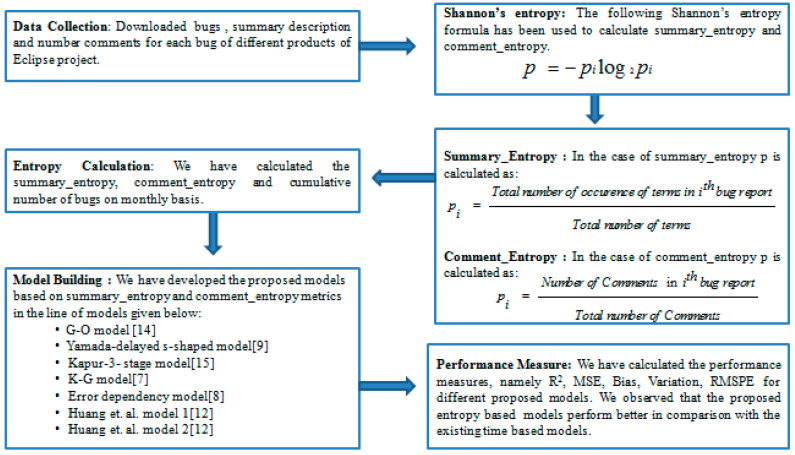
Block diagram of proposed methodology.

The block diagram of the proposed methodology is given in Figure 2.

In the following section, the paper revisits the software reliability growth models which have been used to compare them with the proposed work.

#### 2.4.1. Software Reliability Growth Models (Time vs. Bugs, i.e.,Case 1 in Introduction Section)

In this section, the software reliability growth models, namely G-O model [14],Yamada-delayed S-shaped model [9], Kapur-3-stage model [15], K-G model [7], Error Dependency model [8], Huang et al. model 1 [12] and Huang et al. model 2 [12], have all been revisited. These models have been validated on different products of eclipse project, namely BIRT, CDT, Community, EclipseLink, EMF, Equinox, Orion and Platform.

The models developed in the literature for this study, considered different testing and debugging environments which resulted to the development of models with different mean value functions. These models are based on NHPP property. *x*(*t*) described the cumulative number of bugs detected/fixed in a time interval [0,*t*].

The bug detection/fixing can be described as:

(2) $$\frac{dx(t)}{dt}\propto (y-x(t)),\;or\;\frac{dx(t)}{dt}=g(t)\left(y-x(t)\right),$$

Here, *g*(*t*) is the time dependent fixing rate of bug per remaining bug.SolvingEquation (2), we get the following at time *t* = 0, *x*(0) = 0:

(3) $$x(t)=y\left(1-\exp^{-\int_{0}^{t}g(t)dt}\right)$$

From the above Equation (3), the paper derived different mean value functions depending upon the rate of bug fixing which are given as follows:

**Model 1. G-O model [14]:**

If g(t)=g, Equation (3) reduces to:

(4) x(t)=y(1−exp(−gt))

**Model 2. Yamada-delayed S-shaped model [9]:**

If $g(t)=\frac{g^{2}t}{1+gt},$ Equation (3) reduces to:

(5) x(t)=y(1−(1+gt)exp(−gt))

**Model 3. Kapur-3-stage model [15]:**

If $g(t)=\frac{g^{3}t^{2}}{1+gt+\frac{g^{2}t^{2}}{2}},$ Equation (3) reduces to:

(6) $$x(t)=y\left(1-(1+gt+\frac{g^{2}t^{2}}{2})\exp\left(-gt\right)\right)$$

**Model 4. K-G model [7]:**

If $g(t)=\frac{g}{\left(1+\beta \exp\left(-gt\right)\right)}$, Equation(3) reduces to:

(7) $$x(t)=y\frac{\left(1-\exp(-gt)\right)}{\left(1+\beta \exp(-gt)\right)}$$

During software development, after the design phase of the software, programmers write the code in a programming language to implement it. During the writing of codes, programmers generate different types of errors. Some errors are independent in the sense that they can be removed independently. This means the removal of the errors are not dependent on any other error. There is another category of errors which are known as dependent errors whose removals are dependent on those errors on which they are dependent on, and the fixing of these dependent bugs/errors follow different types of debugging time lag functions denoted by *θ*(*t*). Based on these assumptions, bug dependency models have been proposed in literature as follows [12,13]: software consists of both dependent and independent bugs, hence, the equations for them could be written thus:

(8) $$y=y_{1}+y_{2}$$

where *y*_1_ and *y*_2_ are the number of independent and dependent bugs respectively.

Let *x*(*t*) represent the mean number of bugs removed in time [t,t+Δt]. The fixing of independent and dependent bugs follows an NHPP property:

(9) $$x\left(t\right)=x_{1}\left(t\right)+x_{2}\left(t\right)$$

In the above equation, *x*_1_(*t*) and *x*_2_(*t*) denote the mean value functions of independent and dependent bugs fixed in a time interval. The fixing of independent bugs follows exponential growth curves as these bugs are simple in nature and removed immediately. The following differential equation has thus been written as follows in [14]:

(10) $$\frac{dx_{{}_{1}}\left(t\right)}{dt}=r\times \left[y_{{}_{1}}-x_{{}_{1}}\left(t\right)\right]$$

Solving Equation (10), leads to obtaining the following results at time *t* = 0, x(0)=0.

(11) $$x_{{}_{1}}\left(t\right)=y_{{}_{1}}\left(1-\exp\left(-rt\right)\right)$$

For the dependent bug fixing phenomenon, the following equation has be written in the following manner [8]:

(12) $$\frac{dx_{2}\left(t\right)}{dt}=c\times \left[y_{{}_{2}}-x_{{}_{2}}\left(t\right)\right]\times \frac{x_{{}_{1}}\left(t-\theta \left(t\right)\right)}{y}$$

In the paragraphs that follow, the authors presentthe bug dependency models as proposed in [8,12]. The developed software reliability growth models are based on dependency of the errors and various debugging time lag functions.

**Model 5. Error Dependency Model [8]:**

Here, the following took place:

(13) $$y_{{}_{{}_{1}}}=qy,\;and\;y_{{}_{{}_{2}}}=\left(1-q\right)y,\;\;0\leq q\leq 1$$

Putting the value of *x*_1_(*t*) from Equation (11) in Equation (12) and by taking *θ*(*t*) = 0, we get the following:

(14) $$\frac{dx_{2}\left(t\right)}{dt}=c\times \left[y_{{}_{2}}-x_{{}_{2}}\left(t\right)\right]\times \frac{y_{1}\left(1-\exp\left(-rt\right)\right)}{y}$$

Solving Equation (14) and using Equation (9), the following results were obtained at time $t=0,\;x_{2}\left(0\right)=0$:

(15) $$x\left(t\right)=y\left(1-q\exp\left[-rt\right]-\left(1-q\right)\exp\left[\frac{qc}{r}\left(1-\exp\left[-rt\right]\right)-tqc\right]\right)$$

**Model 6. Huang et al. Model 1 [12]:**

If $\theta \left(t\right)=\left[\frac{1}{r}\log\left(1+rt\right)\right]$ Equation (12) becomes:

(16) $$\frac{dx_{2}\left(t\right)}{dt}=c\times \left[y_{{}_{2}}-x_{{}_{2}}\left(t\right)\right]\times \frac{y_{1}\left(1-\left(1+rt\right)\exp\left(-rt\right)\right)}{y}$$

Solving Equation (16) and using Equation (9), we get the following at time $t=0,\;x_{2}\left(0\right)=0$

(17) $$x\left(t\right)=y\left(1-q\left(1+rt\right)\exp\left[-rt\right]-\left(1-q\right)\exp\left[\frac{2qc}{r}\left(1-\exp\left[-rt\right]\right)-tqc\left(1+\exp\left[-rt\right]\right)\right]\right)$$

**Model 7. Huang et al. Model 2 [12]:**

If $\theta \left(t\right)=\left[\frac{1}{r}\log\left(\frac{\left(\psi +1\right)}{1+\psi \exp\left(-rt\right)}\right)\right]$ Equation (12) becomes:

(18) $$\frac{dx_{2}\left(t\right)}{dt}=c\times \left[y_{{}_{2}}-x_{{}_{2}}\left(t\right)\right]\times \frac{y_{1}\left(1-\exp\left(-rt\right)\right)}{y\left(1+\psi \exp\left(-rt\right)\right)}$$

Solving Equation (14) and using Equation (9), we get the following at time $t=0,x_{2}\left(0\right)=0$

(19) $$x\left(t\right)=y\left(1-q\frac{\left(1+\psi \right)\exp[-rt]}{1+\psi \exp\left[-rt\right]}-\left(1-q\right)\exp\left[-qtc\right]{\left(\frac{1+\psi }{1+\psi \exp\left[-rt\right]}\right)}^{\frac{qc\left(1+\psi \right)}{r\psi }}\right)$$

#### 2.4.2. Entropy-Based Software Reliability Growth Models (Entropy Vs bugs, i.e.,Case 2 and Case 3 in the Introduction Section)

This section proposes a summary and comments of entropy metric-based software reliability growth models in the line of the models proposed in [7,8,9,12,14,15]. The proposed models are based on entropy derived from the bug summary reported by the user and comments submitted by different developers/active users. In this section, the models based on summary and comments have the same functional form as has been made known in the same notation for entropy variable derived from the summary and comments, butthis paper has validated it for the both approaches, i.e.,summary_entropyvs bugs and comment_entropyvs bugs by taking different data sets.

Let x(H(t)) be the mean value function of cumulative number of bugs fixed in entropy interval [0,H(t)] here, entropy is a function of time window. The bug detection/fixing can thus be defined as:

(20) $$\frac{dx(t)/dt}{dH(t)/dt}\propto \left[p-x(H(t))\right],\;or\;\frac{dx(t)/dt}{dH(t)/dt}=k(H(t))(p-x(H(t)))$$

where *k*(*H*(*t*)) is the rate of bug fixed per remaining bug at entropy value *H*(*t*).

Solving above equation with the initial conditions at *t* = 0, H(0)=0 leads to thefollowing:

(21) $$x(t)=p\left(1-\exp^{-\int_{0}^{t}k(H(t))d(H(t))}\right)$$

By taking different values of *k*(*H*(*t*)) in Equation (21), the following proposed models can be derivedbased on these values:

**Model 8:**

If *k*(*H*(*t*)) = *k* Equation(21) reduces to:

(22) x(H(t))=p(1−exp(−kH(t)))

**Model 9:**

If $k(H(t))=\frac{k^{2}H(t)}{1+kH(t)},$ Equation (21) reduces to:

(23) x(H(t))=p(1−(1+kH(t))exp(−kH(t)))

**Model 10:**

If $k(H(t))=\frac{k^{3}H{\left(t\right)}^{2}}{1+kH(t)+\frac{k^{2}H{\left(t\right)}^{2}}{2}},$ Equation (21) reduces to

(24) $$x(H(t))=p\left(1-(1+kH(t)+\frac{k^{2}H{\left(t\right)}^{2}}{2})\exp(-kH(t))\right)$$

**Model 11:**

If $k(H(t))=\frac{k}{\left(1+\delta \exp\left(-kH\left(t\right)\right)\right)},$ Equation (21) reduces to:

(25) $$x(H(t))=p\frac{\left(1-\exp\left(-kH\left(t\right)\right)\right)}{\left(1+\delta \exp\left(-kH\left(t\right)\right)\right)}$$

Let *p*_1_ and *p*_2_ be the proportion of independent and dependent software bugs lying dormant in the software. The following equation where *p* is the sum of both independent and dependent bugs, can be written:

(26) $$p=p_{1}+p_{2}$$

Let x(H(t)) represents the mean number of bugs removed in time [t,t+Δt]. The fixing of independent and dependent bugs follows an NHPP property:

(27) $$x\left(H\left(t\right)\right)=x_{1}\left(H\left(t\right)\right)+x_{2}\left(H\left(t\right)\right)$$

In the above equation, *x*_1_(*H*(*t*)) and *x*_2_(*H*(*t*)) denote the mean value functions of independent and dependent bugs fixed in time interval [0,*t*].

The fixing of independent bugs follows exponential growth curves as these bugs are simple in nature and removed immediately. The following equations could thusemerge from the differential equation:

(28) $$\frac{dx_{{}_{1}}\left(t\right)/dt}{dH\left(t\right)/dt}\propto \left[p_{1}-x_{1}\left(H\left(t\right)\right)\right],\;or\;\frac{dx_{1}(t)/dt}{dH(t)/dt}=l(H(t))(p_{1}-x_{1}(H(t)))$$

Solving Equation (28), The following is ensured at time $t=0,\;x_{1}\left(H\left(0\right)\right)=0$

(29) $$x_{{}_{1}}\left(H\left(t\right)\right)=p_{1}\left(1-\exp\left(-lH\left(t\right)\right)\right)$$

For the dependent bug fixing phenomenon, the equations emanating from it could be written thus:

(30) $$\frac{dx_{2}\left(t\right)/d\left(t\right)}{dH\left(t\right)/d\left(t\right)}=d\times \left[p_{{}_{2}}-x_{{}_{2}}H\left(t\right)\right]\times \frac{x_{{}_{1}}\left(H\left(t\right)-\theta \left(H\left(t\right)\right)\right)}{p}$$

In the following, the bug dependency models are presented. The developed software reliability growth models are based on dependency of the errors and various debugging time lag functions.

Thus, the following steps are taken:

(31) $$p_{{}_{{}_{1}}}=qp,\;and\;p_{{}_{{}_{2}}}=\left(1-q\right)p,\;0\leq q\leq 1$$

**Model 12:**

Putting the value of *x*_1_(*H*(*t*)) from Equation (29) and by taking φ(H(0))=0 in Equation (30), the following ensues:

(32) $$\frac{dx_{2}\left(t\right)/d\left(t\right)}{dH\left(t\right)/d\left(t\right)}=d\times \left[p_{{}_{2}}-x_{{}_{2}}\left(H\left(t\right)\right)\right]\times \frac{p_{1}\left(1-\exp\left(-lH\left(t\right)\right)\right)}{p}$$

Solving Equation (32) and using Equation (27), the authors arrive at the following at time $t=0,\;x_{2}\left(H\left(0\right)\right)=0$

(33) $$xH\left(t\right)=p\left(1-q\exp\left[-lH\left(t\right)\right]-\left(1-q\right)\exp\left[\frac{qd}{l}\left(1-\exp\left[-lH\left(t\right)\right]\right)-H\left(t\right)qd\right]\right)$$

The following models are developed using different debugging time lag functions.

**Model 13:**

If $\theta \left(H\left(t\right)\right)=\left[\frac{1}{l}\log\left(1+lH\left(t\right)\right)\right],$ Equation (30) reduces to:

(34) $$\frac{dx_{2}\left(t\right)/d\left(t\right)}{dH\left(t\right)/d\left(t\right)}=d\times \left[p_{{}_{2}}-x_{{}_{2}}H\left(t\right)\right]\times \frac{p_{1}\left(1-\left(1+lH\left(t\right)\right)\exp\left(-lH\left(t\right)\right)\right)}{p}$$

Solving Equation (34) and using Equation (27), the following results emerge at time $t=0,\;x_{2}\left(H\left(0\right)\right)=0$

(35) $$xH\left(t\right)=p\left(1-q\left(1+lH\left(t\right)\right)\exp\left[-lH\left(t\right)\right]-\left(1-q\right)\exp\left[\frac{2qd}{k}\left(1-\exp\left[-lH(t)\right]\right)-H(t)qd\left(1+\exp\left[-lH(t)\right]\right)\right]\right)$$

**Model 14:**

If $\theta \left(H\left(t\right)\right)=\left[\frac{1}{l}\log\left(\frac{\left(\psi +1\right)}{1+\psi \exp\left(-lH\left(t\right)\right)}\right)\right]$, Equation (30) reduces to:

(36) $$\frac{dx_{2}\left(t\right)/d\left(t\right)}{dH\left(t\right)/d\left(t\right)}=d\times \left[p_{{}_{2}}-x_{{}_{2}}\left(H\left(t\right)\right)\right]\times \frac{p_{1}\left(1-\exp\left(-lH\left(t\right)\right)\right)}{p\left(1+\psi \exp\left(-lH\left(t\right)\right)\right)}$$

Solving Equation (36) and using Equation (27), the following results emerge at time $t=0,\;x_{2}\left(H\left(0\right)\right)=0$:

(37) $$xH\left(t\right)=p\left(1-q\frac{\left(1+\psi \right)\exp[-lH\left(t\right)]}{1+\psi \exp\left[-lH\left(t\right)\right]}-\left(1-q\right)\exp\left[-qH\left(t\right)d\right]{\left(\frac{1+\psi }{1+\psi \exp\left[-kH\left(t\right)\right]}\right)}^{\frac{qd\left(1+\psi \right)}{pl\psi }}\right)$$

The value of parameters for the models (model 1 to model 14) have been estimated for different products of the Eclipse project. The values of parameters *y* and *p* give the potential number of bugs lying dormant in the software. x(t) and x(H(t)) gives the number of bugs fixed. The differences y−x(t) and p−x(H(t)) indicate the number of bugs still lying in the software and this quantity determines the reliability level of the software. It is the quantitative quality measurement of the software product. The parameter value *q* indicates the proportion of independent bugs. With the help of this parameter, we can find the proportion of independent and dependent bugs still lying dormant in software. The values of *β* and *ψ* give the nature of the growth curves. A high value indicates that the bugs are complex and will take more time in fixing. The bug fixing rate tells us about the bug fixing efficiency.

## 3. Results and Analysis

So far, the authors have validated the proposed summary_entropy and comment_entropy-based software reliability growth models on eight datasets, namely the BIRT, CDT, Community, EclipseLink, EMF, Equinox, Orion and Platform products of Eclipse project. In our study, we have developed three cases, namely calendar time vs. bugs (case1), summary_entropyvsbugs(case2) and comment_entropyvsbugs(case3). The paper identified a total number of 168 cases, i.e., eight datasets *three cases* seven models. The paper has estimated the unknown parameters of the models using the Statistical Package for Social Sciences Software (SPSS, *https://www.ibm.com/products/spss-statistics*) and tabulated the results in Table 2, Table 3, Table 4, Table 5, Table 6, Table 7, Table 8 and Table 9. The performance measures, namely *R*^2^, *MSE, Bias, Variance* and *RMSPE* of all the models have been tabulated in Table 10, Table 11, Table 12, Table 13, Table 14, Table 15, Table 16 and Table 17. In the performance table, the bold value indicates the maximum value of *R*^2^ across all the cases for all the products.

A comparison of case 1 and case 2, indicates that case 2 gives better performance in 44 cases while case 1 gives only 10 cases. A comparison of both cases reveals that in two cases both cases have equal performance. Model 13 and model 14 outperforms across all the products in case 2 in comparison with case 1.

A comparison of case 1 and case 3, reveals that case 3 gives a better performance than case 1 in 48 cases in only eight cases for all the products of Eclipse project. Model 11, model 12, model 13 and model 14 outperform across all the products in case 3 in comparison with case 1.

Comparing cases 2 and 3, reveals that case 3 gave better performance in 25 cases whilecase 2 gives better performance in 20 cases. In all, in11 cases both the cases gave equal performance.

The study discovered that the models depending on calendar time, summary_entropy, and comment_entropy performed better in 18, 64and 73 cases, respectively, out of 168 cases in terms of various performance measures. We can conclude that the entropy-based models perform best in 137 out of 168 cases.

Summary_entropy metric-based proposed models have performed better in 78.57% cases and Comment_entropy metric-based proposed models performed better in 85.71% cases in comparison with time-based models. Comment_entropy-based proposed models performed better in 44.64% cases in comparison with summary_entropy-based proposed models. The authors conclude that entropy-based proposed models outperform in 81.55% cases. We also observed that in the cases, where case 1 i.e., time-based models perform better, it overestimated the value of the potential number of bugs. The authors concluded that the entropy-based proposed models (model 8 to 14) performed significantly better in comparison with time-based models (model 1 to 7).

The results analysis arising from the table of performance measures for the different products reviewed for the study revealed that, model 11 of case 3, model 11 of case 2 andcase 3, model 11 of case 2, model 14 of case 3, model 14 of case 3, give the best performance for the BIRT, Community, EclipseLink, EMF and Platform products, respectively. For the CDT and Equinox products model 11 of case 2 and case 3, model 12 of case 2 and model 14 of case 2 and case 3, gave the best performance. For the Orion product model 13 and model 14 of case 3 gave the best performance.

The authors have so far presented the goodness of fit curves of proposed models in Figure 3, Figure 4, Figure 5, Figure 6, Figure 7, Figure 8, Figure 9 and Figure 10 for case 2 and Figure 11, Figure 12, Figure 13, Figure 14, Figure 15, Figure 16, Figure 17 and Figure 18 for case 3. It was observed that the predicted values of the proposed models were very close to the observed value. The proposed models exhibit better goodness of fit in most of the cases in comparison with the existing models.

## 4. Related Work

The proposed work in this paper deals with the mathematical modeling based on two types of entropy: summary and comments entropy. During the evolution of the software products, a reported bug is assigned to a contributor/developer who can fix this bug. This process is regarded as bug triaging. Bug triaging is one of the important parts of the bug fixing process. During fixing of bugs, it was observed that bugs lie in two categories: independent and dependent bugs. Independent bugs are those bugs which can be fixed independently, but dependent bugs are those whose fixing isdependent on fixing other bugs on which they are dependent. The proposed models in this paper considered bug dependency during theprocess of fixing bugs. It is also dependent on summary and comment entropy metrics.The authors of this paper have organized the related work of the paper in five sections. Section 4.1 deals with bug triaging. Section 4.2 describes the summary description and how these textual descriptions were used in developing bug severity and priority prediction models. During the bug fixing processes, different contributors submitted the comments which help in bug fixing. Bug comments are discussed in Section 4.3. Section 4.4 describessoftware reliability growth models available in the literature. Section 4.5 presentshow entopyisused in developing prediction models.

### 4.1. Bug Triaging

The purpose of bug triaging is to assign a bug to suitable or appropriatedevelopers. The bug fixing process is a crucial task to reduce time and efforts. In a previousstudy [24], theauthors demonstrated how to assign bug reports to developers automatically by using text categorization. One such experiment was empirically validated on 15,859 bug reports of the Eclipse datasets. The authors used machine learning techniques, aNaive Bayes classifierand obtained results with 30% accuracy.Later on, Anvik et al. [25] extended the work of Cubranic and Murphy [24] by applying different classification techniques namely, NB, SVM and C4.5. The empirical investigation was conducted on 8655 bug reports for Eclipse and 9752 for Firefox. The authors achieved aprecision of 64% and 57% for the Firefox and Eclipse datasets, respectively. In [26], the authors proposed a new approach to assist bug triagers in open source software projects, through a semi-automated bug assignment process. Experimental results wereconducted on 5200 bug reports of the Eclipse JDT project and achieved an average precision and recall of 90.1% and 45.5%, respectively. An attempt has been made in [27] using a NB technique with bug assignment graphs and incremental learning. The empirical investigation was conducted on 856,259 bug reports of the Eclipse and Mozilla projects and achieved the prediction accuracy up to 86.09%.

An attempt has been made to propose a new approach called Bugzie for automatic bug triaging using fuzzy sets and a cache-based automatic approach [28]. Bugzie believes that fuzzy set-software systems are associated with every technical term. A fuzzy set is used to indicate that the developer is correcting the bugs associated with each term. The value of the membership function of the fuzzy set is obtained from the bug reports that has been corrected and updated when the newly fixed bug report is available.

To find the most appropriate developer for newly incoming bug reports, based on the technical terms, Bugzie combines fuzzy sets and classifies developers based on the values of member functions.In [29], the authors proposed several techniques such as intra-fold updates and refined classification.The experimental results were validated on 856,259 bug reports of the Mozilla and Eclipse projects. The authors reduced the length of tossing path by the prediction accuracy of 83.62%. Effortshave been made to develop automatic approaches to predict an appropriate developer with admissible experience to solve the newly coming bug reports in order to reduce time, effort and cost in bug triaging [30]. In another similar study [31], the authors proposed Markov chains using a graph model in order to capture bug tossing history and reducing tossing events, by upto72%.The experimental results were validated on 445,000 bug reports and achieved prediction accuracy ofup to 23%. In [32], a semi-supervised text classification for bug triaging process was proposed. The authors used a Naïve Bayes classifier and improved the accuracy by 6%. In a study by [33], the data scale used for the study used werereduced by using data reduction techniques in bug assignment process. The experimental result was validated on the Eclipse open source project which achieved 96.5% accuracy inbug triaging, which was better than the existing work. In [34], the authors used various reduction techniques for an effective bug triaging. The experimental result of data reduction was validated on 600,000 bug reports of the Mozilla and Eclipse projects. In [35], the authors presented a unified model that combineswiththe previous activity information from the developer with the location of suspicious programs with respect to bug reporting in the form of similar functions. The authors demonstrated how this works on more than 11,000 bug reports. The proposed work gavebetter results in comparison withAnvik et al. [36] and Shokripour et al. [37]. Goyal and Sardana [38] proposed a new bug triaging approach, W8Prioritizer, which is based on the priority of bug parameters. The authors expand the study of triaging for non-reproducible (NR) bugs. When a developer encounters a problem in reproducing a bug report, he/she marks the bug report as NR. However, some parts of these bugs are reproduced and eventually fixed later. In order to predict the fixability of bug reports marked as NR, a prediction model, NRFixer, has been proposed. It is evident from literature review for this study that fixing/removal of bugs have been efficiently and automatically managed by bug triager.It is evident from the literature survey that triaging is based on machine learning techniques and it has not used any models which considers dependency and prediction of bugs in a time window.

### 4.2. Prediction Modeling Based on Bug Summary Metric

The summary attribute contains bug report descriptions. It plays the major role in prediction of severity and priority of reported bugs. In [39], the authors presented a reliable approach to predict the bug severity of newly incoming bug reports labeled as normal. The experimental result was conducted on Eclipse and Mozilla datasets and gave an improved result. A classification technique based on a text called concept profile to assess the severity levels of reported bug has been proposed in [40]. The authors evaluated and compared their approach with three classifications algorithms, namelyKNN, NB and NBM. The empirical investigation was conducted on Eclipse and Mozilla Firefox datasets and the evaluated result performed better. In [41], the authors proposed a text mining approach using the NB machine learning technique for predicting the severity level of bugs. The experimental resultswasbasedonthe Mozilla and Eclipse projects.The authors revealed that the introduction of bi-grams can improve the accuracy, but in some cases, it can worsen it. Another attempt was made by Chaturvediand Singh [42] to compare the performance of different machine learning techniques, namely SVM, NB, KNN, NBM, J48 and RIPPER for predicting the bug severity level of a newly incoming bug report.

A new way to retrieve information based on the similarity function of the BM25 document to automatically predict the severity of reported bugs was proposed in [43]. In [44], the authors used a NB machine learning technique with different feature selection schemes.The experimental result was conducted on the Mozillaand Eclipse projects. Chaturvedi and Singh [45] used different machine learning algorithms to predict the severity of newly incoming bug reports. The empirical investigation was conducted on data of NASA from the PROMISE repository using a textual description of bug reports. In [46], the authors predicted the severity of newly incoming bug reports by analyzing theirtextual descriptions using text mining algorithms. The approach has been validated on the Mozilla, Eclipse and GNOME projects. This study has been extended by Lamkanfi et al. [47] to compare with a few other data mining algorithms such as NBM, KNN and SVM to predict bug severity for newly incoming bug reports. The authors concluded that NBM outperforms the other data mining algorithms.

In [48], the authors proposed a new and automated approach called SEVERityISsue assessment (SEVERIS), which predicts the severity levels of defect reports. In [49], the authors used different machine learning techniques, namely, Naïve Bayes, Decision Tree and Random Forest for bug priority prediction. The authors introduced two feature setsin theclassification accuracy. The result was validated on the Eclipse, Firefox datasets and shows that feature-set-2outperforms feature-set-1.Kanwal et al. [50] proposed a bug priority recommender which is developed by using SVM classification techniques. The bug priority recommender is used to automatically assign a priority level to newly incoming bugs. The authors validated the result on platform products of the Eclipse dataset. This study has since been extended by Kanwal et al. [51] who compared which classifier performs better in terms of accuracy. The result shows that SVM performance is better than the Naïve Bayes for textual features and Naïve Baiyes is better than SVM for categorical features. In [52], the authors have evaluated the performance of different machine learning techniques, namely, Support Vector Machine (SVM), Naïve Bayes (NB), k-Nearest Neighbor (KNN) and Neural Network (NNet) by using summary attributes to predict the bug priority of newly incoming bug reports. The accuracy of different machine learning techniques in predicting the priority of a reported bug within and across projects was found above 70%, except for the Naïve Bayes technique.

It is evident from the literature survey related to a summary description of a reported bug that summary description plays an important role in bug severity prediction and hence, assist in bug fix scheduling. In all these works, the textual description of the summary has been taken for the study. The authors in this paper, moved a step forward and developed an entropy-based summary description metric (summary_entropy) to develop mathematical models.

### 4.3. Bug Comments

During the bug fixing process, different contributors attached various comments as solutions to fix the bug. The comments submitted by the developers/active users assisted in the bug fixing process. The textual description of users’ comments can affect the comprehensibility of bug report, so it is important to measure them. In [53], the authors proposed an approach to measure the textual coherence of the user comments in bug reports. The results were validated on the Eclipse project and suggest that the proposed measure correlates with assessments provided by software developers. Xuan et al. [54] proposed issues recommended by the commenters as a multi-label recommendation task which improves the cooperation between the developer and the bug content in order to see the corresponding commenters. The recalled value was found between 41% and 75% for top-10 recommendation. In this paperhowever, the authors haveconsidered the uncertainties associated with the number of comments in terms of entropy and used this entropy-based bug comments metric (comment_entropy) to develop mathematical models.

### 4.4. NHPP-Based Software Reliability Growth Modeling

From available literature considered for this study, it has been observed that authors have proposed software reliability growth models that could provide quantitative indicators for a software performance prediction. The reliability is an important quality attribute of software product [1]. An effort was made by Goel and Okumoto [14] to develop an NHPP-based model for an error removal process. The developed model mean value function followed an exponential growth curve. Yamada et al. [9] proposed an S-shaped model foran error removal phenomenon. The authors assumed that the detected fault cannot be immediately removed.An attempt was made by Kapur et al. [7] to develop an NHPP-based SRGM. In [8], the authors developed a software reliability growth model that focused on the underlying error dependencies in software systems. A model has been proposed to take care of faults of complex nature where they are detected, isolated and then removed [15].

A number of testing effort and testing coverage dependent SRGMs have been proposed in the literature [16,17,18,19,20,49,50,52,55]. The software reliability modelsincorporatingthe dependent faults concept with fixing time/debugging time lag have been proposed in [12]. The authors show that fault dependency and debugging time lag-based software reliability models have an accurate prediction capability. Singh et al. [13] proposed several SRGMs based on the power function of execution time of the software. The authors were able to show that the proposed SRGM models based ondependent fault and fixing time lag with execution time as a power functionprovided fairly accurate predictions. Kapur et al. [10] proposed an SRGM by considering change-point and effort control with execution time as a power function of time. The proposed work provides a solution to project managers toget the desired reliability level. In [11], the authors developed a class of SRGM by considering testing time execution as a power function. Mean Squared Error (MSE) was used as the measure of ‘goodness of fit’. The authors show that the results are fairly accurate and close to the observed values. In [56], the authors proposed two-dimensional SRGM, whichweredescribed as an SRGM based on testing-time and testing-effort functions. Kapur et al. [57] proposed a two dimensional software reliability growth model which consists of testing time and testing coverage.

In another study [58], the authors proposed a stochastic model based on an NHPP for software failure phenomena. From the available literature on this subject, it was observed that the models developed were based on calendar time, testing coverage and testing effort functions. In this paper however, the authors strove to developed models based on summary and comment entropy which has been proven to be a novel approach in the sense that, it considers bug fixing as a function of summary and comment.

### 4.5. Entropy-Based Prediction Modeling

Studies in this area revealed that attempts have been made by Hassan [2] to propose code change metrics. The empirical investigation was conducted in change history of six large open source projects. The author shows that the proposed approach performs better in comparison to the other historical predictions of bugs. In another study [4], the authors proposed an entropy-based modeltomeasure the uncertainty arising due to source code changes in the software product. The experimental result was conducted on seven components of the Mozilla project. From this premise, it was observed that for all the components, the value of *R*^2^ was found to be more than 0.95. In another study [5], the authors proposed an approach that predicted the potential number of bugs by considering: (i) traditional SRGM, (ii) entropy-based models and (iii) potential bugs based on entropy. In thestudy, it was observed that the potential complexity of code change(entropy)-based approach wasbetter. Instudy [59], the authors proposed entropy-based software reliability analysis. The experimental result was validated on five products of the Apache open source projects, namely Avro, Pig, Hive, jUDDI and Whirr. In [58] the authors proposed an entropy optimized Latent Dirichlet Allocation (LDA) approach for bug assignment. The experimental results were validated on the Eclipse JDT and Mozilla Firefox projects and recallsofup to 84% and 58% were achieved, and precisionsof ofup to 28% and 41%, respectively. Recently, the authors developed entropy-based regression models to predict the bad smells [6].

The complexity of code changes/entropy available in the literature is based on the code change process of the software. In [60], a joint entropy model was used to reduce the possibility of double, useless, and even wrong cases. Thereafter, a database was created that used a large number of photographs. The full database-based experiment demonstrates that the model’s superiority is that the author’s model can not only reduce the number of learning instances, but also maintain the accuracy of the retrieval.In this paper however, the authors have developed two new metrics, i.e., summary_entropy and comment_entropy, based on the bug summary descriptions reported by users and comments submitted by developers to developed bug-based SRGM.

## 5. Conclusions

In this study so far, efforts were made to proposed novel approach for developing software reliability growth models. The paper considered the summary description of a bug reported by users on a bug tracking system and the number of comments submitted by active users/developers during the bug fixing process.The paper also quantified the value of summary description and comments in terms of entropy which also measured the uncertainty arisingas a result of the enormous size of bug repositories and irregularity on the bug tracking system. The authors of this paper thus developed the models of different nature, ranging from exponential to S-shaped or mixture of both, depending larglyon summary and comments metrics. Bug dependencies are always present in software due to errors in code writing, misunderstanding of users requirements and faults in software architecture.The developed models considered bug dependency with different delay-effect factors in debugging. By this, the authors validated the proposed models on eight products of the Eclipse project, namely BIRT, CDT, Community, EclipseLink, EMF, Equinox, Orion and Platform. The proposed models were compared with the existing time-based models. Here, it was observed that the models based on calendar time, summary_entropy, and comment _entropy perform better in 18, 64 and 73 cases, respectively, out of 168 cases in terms ofthe performance measures *R*^2^, variation and RMSPE. Summary entropy metric-based proposed modelswere observed to have performed better in 78.57%of the cases in comparison with time-based models. Comment entropy metric-based proposed models performed better in 85.71% cases in comparison withtime-based models.We also observed that in the cases, where case 1, i.e., t vs. bugs performs better, it overestimated the value of the potential number of bugs. From this premise, the authors concluded that the proposed models performed significantly better in comparison with t vs. bugs models (model 1 to 7). It also provided an optimal value of potential bugs. In the future, further work could be done in the area of the summary_entropy and comment_entropymetric-based models using other project data to make it general.

## Figures and Tables

**Figure 3:**
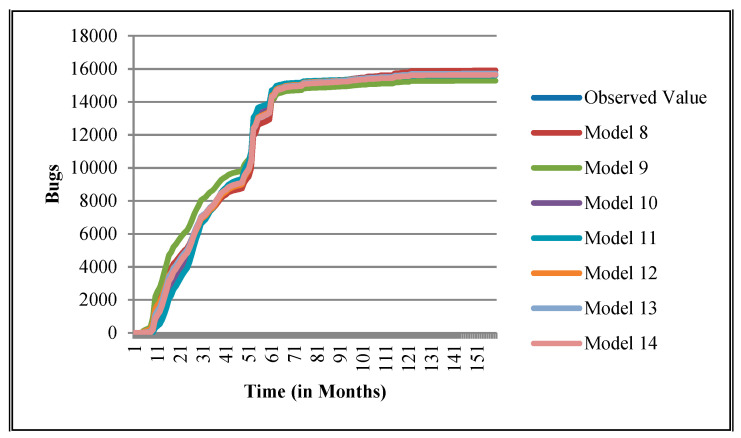
Goodness of fit curves of proposed models for BIRT.

**Figure 4:**
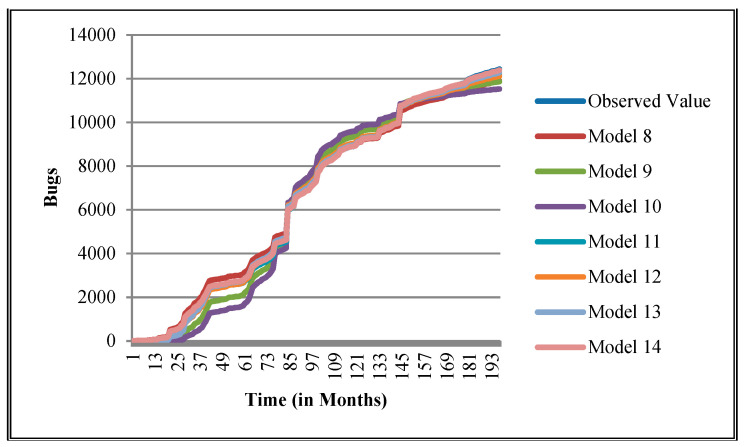
Goodness of fit curves of proposed models for CDT.

**Figure 5:**
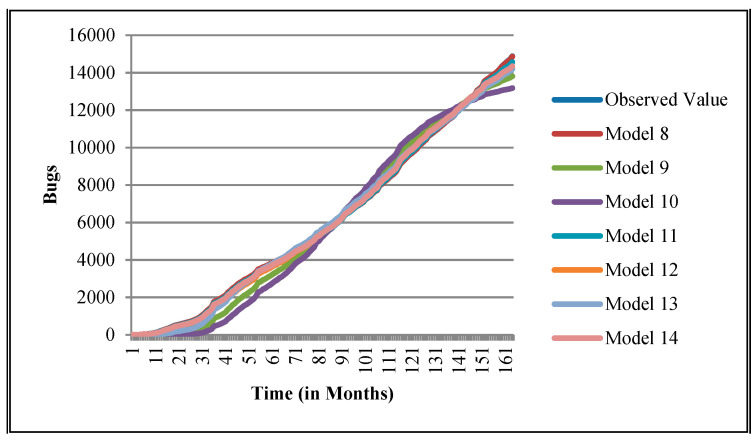
Goodness of fit of curves proposed models for Community.

**Figure 6:**
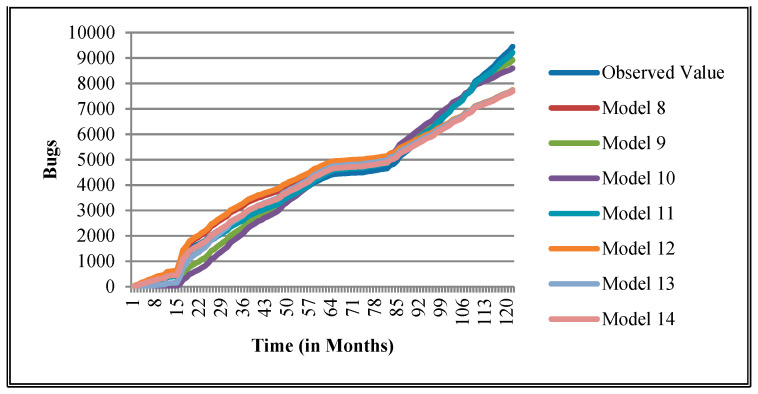
Goodness of fit curves of proposed models for EclipseLink.

**Figure 7:**
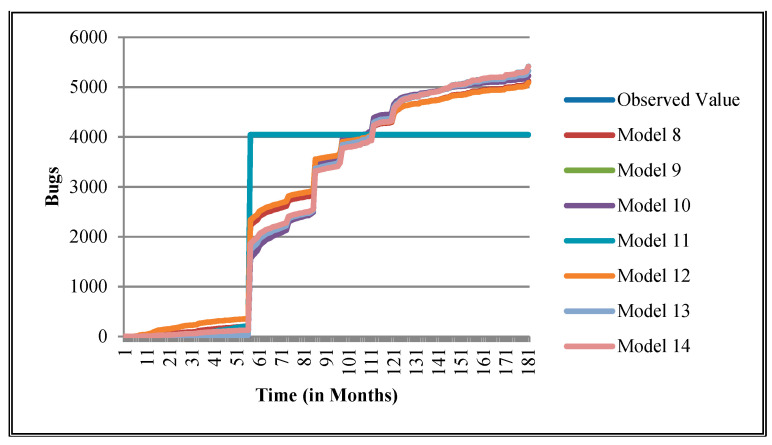
Goodness of fit curves of proposed models for EMF.

**Figure 8:**
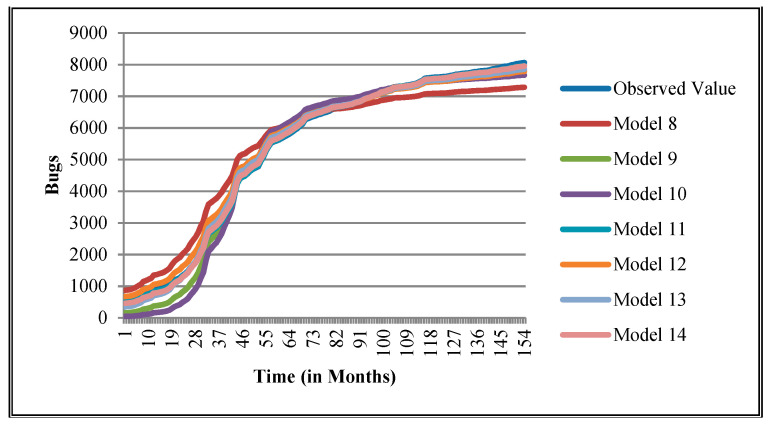
Goodness of fit curves of proposed models for Equinox product.

**Figure 9:**
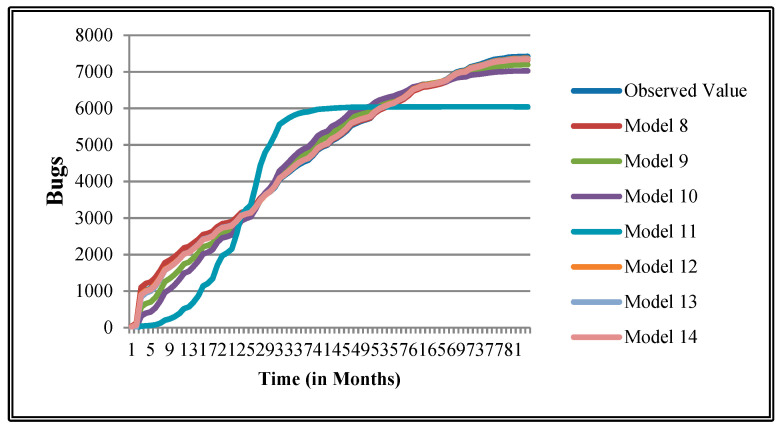
Goodness of fit curves of proposed models for Orion.

**Figure 10:**
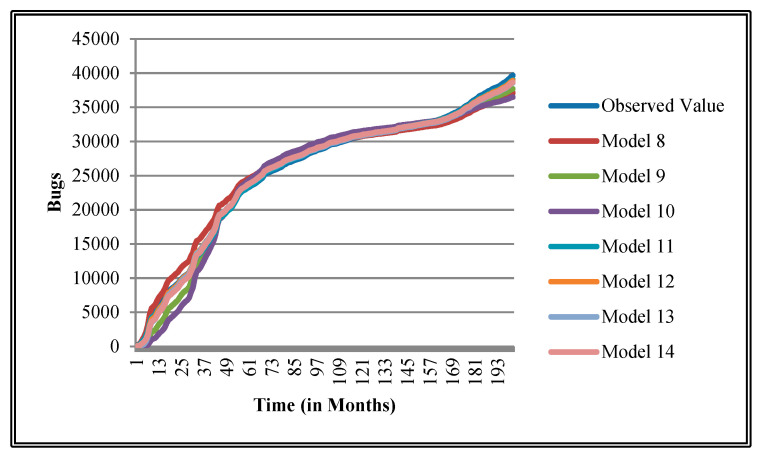
Goodness of fit curves of proposed models for Platform.

**Figure 11:**
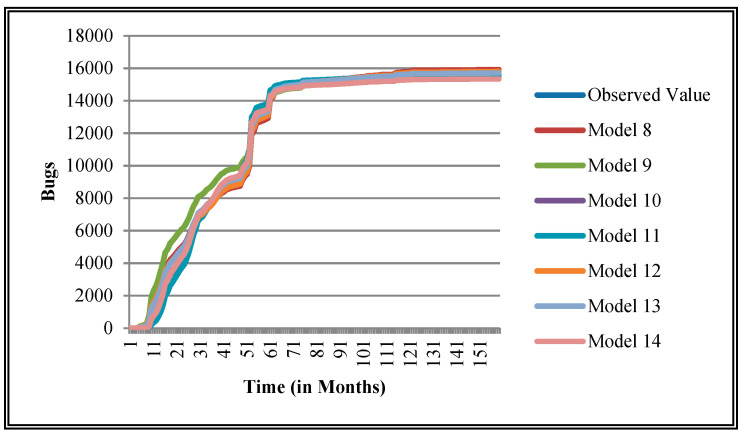
Goodness of fit curves of proposed models for BIRT.

**Figure 12:**
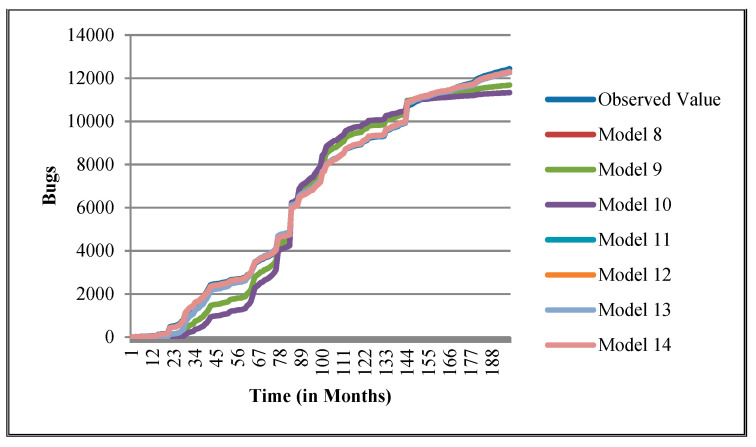
Goodness of fit curves of proposed models for CDT.

**Figure 13:**
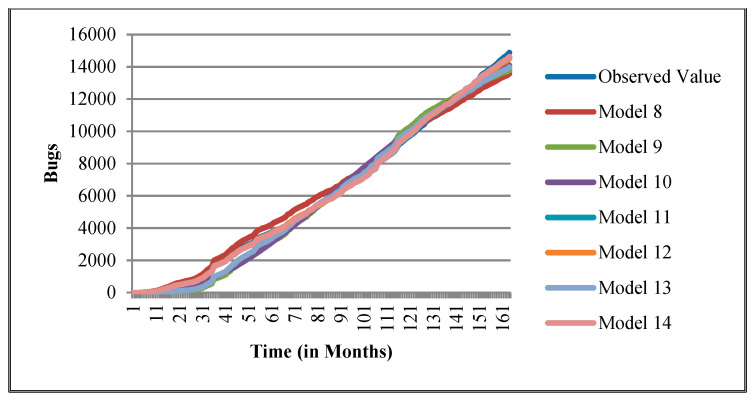
Goodness of fit curves of proposed models for Community.

**Figure 14:**
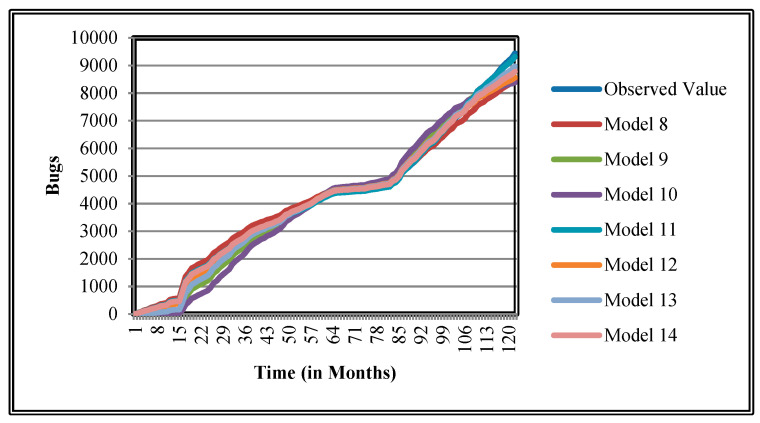
Goodness of fit curves of proposed models for EclipseLink.

**Figure 15:**
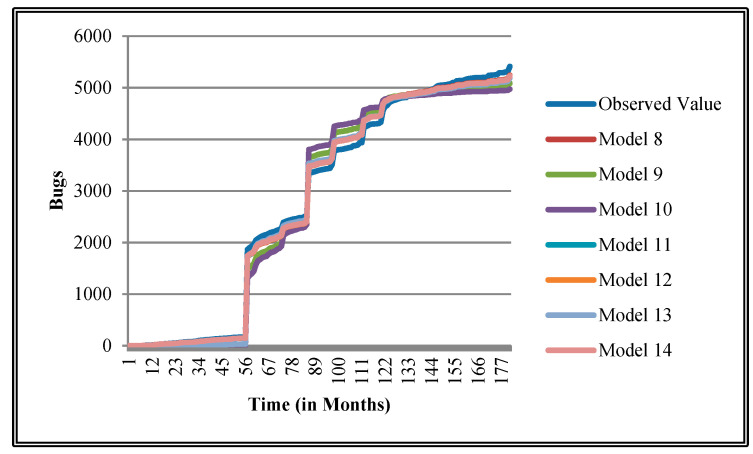
Goodness of fit curves of proposed models for EMF.

**Figure 16:**
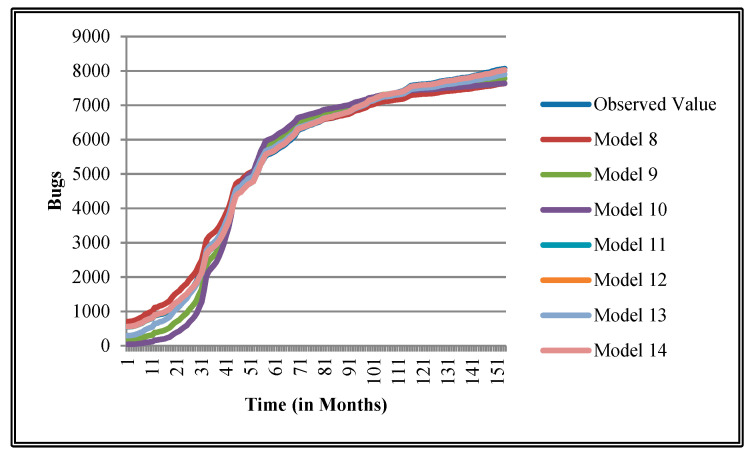
Goodness of fit curves of proposed models for Equinox.

**Figure 17:**
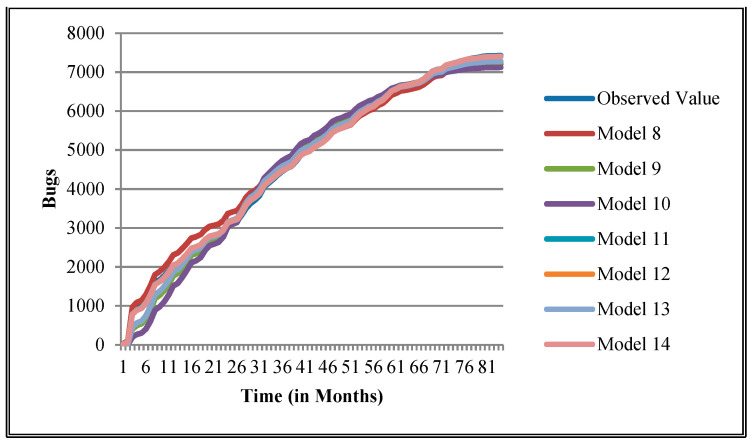
Goodness of fit curves of proposed models for Orion.

**Figure 18:**
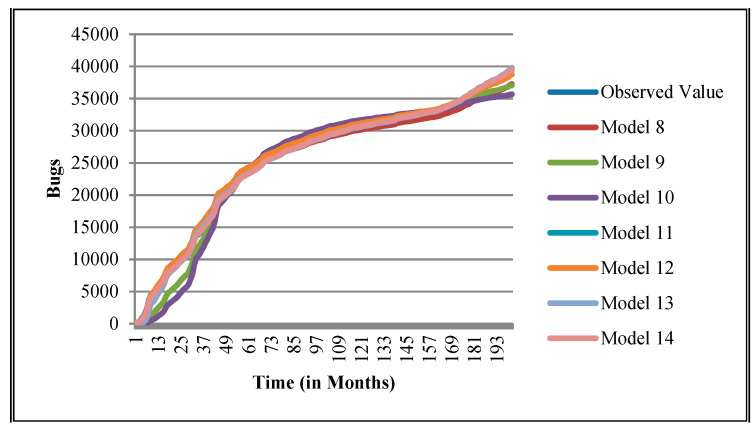
Goodness of fit curves of proposed models for Platform.

**Table 1:** Number of bug reports in each product of Eclipse project.

Product	Number of Bugs	Observation Period
BIRT	15,914	January2005–May 2018
CDT	12,438	January 2002–June 2018
Community	14,881	March 2002–June 2018
EclipseLink	9447	March 2002–June 2018
EMF	5413	September 2002–June 2018
Equinox	8066	October 2001–June 2018
Orion	7425	December 2010–June 2018
Platform	39,434	October 2001–June 2018

**Table 2:** Parameter Estimates for BIRT.

Models	Prediction Classes	Actual Bugs	Parameter Estimates						
			*y*	*g*	*c*	*q*	*r*	*ψ*	*β*
Model 1	Case 1	15,914	17,831	0.019	-	-	-	-	-
Model 8	Case 2		26,390	0.001	-	-	-	-	-
Model 8	Case 3		27,393	0.060	-	-	-	-	-
Model 2	Case 1		16,302	0.047	-	-	-	-	-
Model 9	Case 2		20,305	0.002	-	-	-	-	-
Model 9	Case 3		20,398	0.205	-	-	-	-	-
Model 3	Case 1		15,923	0.074	-	-	-	-	-
Model 10	Case 2		17,427	0.004	-	-	-	-	-
Model 10	Case 3		17,489	0.381	-	-	-	-	-
Model 4	Case 1		16,018	0.056	-	-	-	-	5.473
Model 11	Case 2		26,364	0.001	-	-	-	-	1.966
Model 11	Case 3		24,895	0.136	-	-	-	-	2.166
Model 5	Case 1		16,230	-	0.373	0.168	0.021	-	-
Model 12	Case 2		26,581	-	0.028	0.024	0.020	-	-
Model 12	Case 3		22,901	-	0.561	0.202	0.183	-	-
Model 6	Case 1		16,125	-	0.074	0.677	0.053	-	-
Model 13	Case 2		28,425	-	0.011	0.062	0.009	-	-
Model 13	Case 3		18,254	-	0.439	0.418	0.334	-	-
Model 7	Case 1		16,143	-	0.180	0.321	0.035	1.000	-
Model 14	Case 2		28,485	-	0.020	0.032	0.009	0.992	-
Model 14	Case 3		28,131	-	0.617	0.117	0.244	0.000	-

**Table 3:** Parameter Estimates for CDT.

Models	Prediction Classes	Actual Bugs	Parameter Estimates						
			*y*	*g*	*c*	*q*	*r*	*ψ*	*β*
Model 1	Case 1	12,438	20,039	0.005	-	-	-	-	-
Model 8	Case 2		29,190	0.001	-	-	-	-	-
Model 8	Case 3		22,449	0.061	-	-	-	-	-
Model 2	Case 1		16,437	0.015	-	-	-	-	-
Model 9	Case 2		14,343	0.003	-	-	-	-	-
Model 9	Case 3		12,697	0.318	-	-	-	-	-
Model 3	Case 1		13,601	0.028	-	-	-	-	-
Model 10	Case 2		12,365	0.006	-	-	-	-	-
Model 10	Case 3		11,629	0.549	-	-	-	-	-
Model 4	Case 1		12,591	0.031	-	-	-	-	15.207
Model 11	Case 2		18,114	0.002	-	-	-	-	2.024
Model 11	Case 3		22,114	0.063	-	-	-	-	0.016
Model 5	Case 1		13,907	-	0.580	0.058	0.005	-	-
Model 12	Case 2		26,113	-	0.011	0.057	0.015	-	-
Model 12	Case 3		24,188	-	0.094	0.476	0.120	-	-
Model 6	Case 1		13,717	-	0.160	0.114	0.027	-	-
Model 13	Case 2		25,343	-	0.026	0.026	0.024	-	-
Model 13	Case 3		26,486	-	0.376	0.119	1.000	-	-
Model 7	Case 1		20,952	-	0.206	0.030	0.035	1.00	-
Model 14	Case 2		29,598	-	0.004	0.996	0.001	0.98	-
Model 14	Case 3		22,034	-	0.069	0.918	0.067	0.00	-

**Table 4:** Parameter Estimates for Community.

Models	Prediction Classes	Actual Bugs	Parameter Estimates						
			*y*	*g*	*c*	*q*	*r*	*ψ*	*β*
Model 1	Case 1	14,880	18,764	0.006	-	-	-	-	-
Model 8	Case 2		21,459	0.001	-	-	-	-	-
Model 8	Case 3		25,765	0.057	-	-	-	-	-
Model 2	Case 1		36,742	0.008	-	-	-	-	-
Model 9	Case 2		15,904	0.003	-	-	-	-	-
Model 9	Case 3		16,347	0.250	-	-	-	-	-
Model 3	Case 1		20,035	0.022	-	-	-	-	-
Model 10	Case 2		13,751	0.006	-	-	-	-	-
Model 10	Case 3		13,945	0.468	-	-	-	-	-
Model 4	Case 1		25,352	0.017	-	-	-	-	15.207
Model 11	Case 2		22,653	0.002	-	-	-	-	1.440
Model 11	Case 3		31,681	0.079	-	-	-	-	1.169
Model 5	Case 1		21,790	-	0.174	0.046	0.017	-	-
Model 12	Case 2		21,125	-	0.009	0.129	0.004	-	-
Model 12	Case 3		27,363	-	0.193	0.445	0.094	-	-
Model 6	Case 1		25,043	-	0.273	0.032	0.028	-	-
Model 13	Case 2		24,922	-	0.024	0.035	0.022	-	-
Model 13	Case 3		26,304	-	0.237	0.233	0.472	-	-
Model 7	Case 1		20,385	-	0.105	0.068	0.029	0.971	-
Model 14	Case 2		19,304	-	0.003	0.755	0.002	1.000	-
Model 14	Case 3		28,628	-	0.089	0.750	0.104	0.993	-

**Table 5:** Parameter Estimates for EclipseLink.

Models	Prediction Classes	Actual Bugs	Parameter Estimates						
			*y*	*g*	*c*	*q*	*r*	*ψ*	*β*
Model1	Case 1	9447	9308	0.011	-	-	-	-	-
Model 8	Case 2		15,517	0.001	-	-	-	-	-
Model 8	Case 3		15,552	0.062	-	-	-	-	-
Model 2	Case 1		11,259	0.021	-	-	-	-	-
Model 9	Case 2		13,609	0.980	-	-	-	-	-
Model 9	Case 3		10,221	0.270	-	-	-	-	-
Model 3	Case 1		8791	0.042	-	-	-	-	-
Model 10	Case 2		10,175	0.006	-	-	-	-	-
Model 10	Case 3		8814	0.498	-	-	-	-	-
Model 4	Case 1		28,848	0.008	-	-	-	-	2.952
Model 11	Case 2		19,976	0.002	-	-	-	-	3.756
Model 11	Case 3		19,678	0.077	-	-	-	-	0.866
Model 5	Case 1		9469	-	0.147	0.107	0.030	-	-
Model 12	Case 2		14,495	-	0.064	0.016	0.000	-	-
Model 12	Case 3		10,343	-	0.746	0.254	0.210	-	-
Model 6	Case 1		16,686	-	0.080	0.071	0.099	-	-
Model 13	Case 2		12,320	-	0.048	0.030	0.021	-	-
Model 13	Case 3		18,645	-	0.249	0.197	0.534	-	-
Model 7	Case 1		9574	-	0.064	0.512	0.016	0.723	-
Model 14	Case 2		11,849	-	0.012	0.896	0.002	1.000	-
Model 14	Case 3		11,609	-	0.207	0.681	0.187	0.955	-

**Table 6:** Parameter Estimates for EMF.

Models	Prediction Classes	Actual Bugs	Parameter Estimates						
			*y*	*g*	*c*	*q*	*r*	*ψ*	*β*
Model1	Case 1	5413	8327	0.005	-	-	-	-	-
Model 8	Case 2		10,924	0.001	-	-	-	-	-
Model 8	Case 3		13,177	0.042	-	-	-	-	-
Model 2	Case 1		7767	0.015	-	-	-	-	-
Model 9	Case 2		7415	0.999	-	-	-	-	-
Model 9	Case 3		5913	0.288	-	-	-	-	-
Model 3	Case 1		6228	0.029	-	-	-	-	-
Model 10	Case 2		6045	0.010	-	-	-	-	-
Model 10	Case 3		5249	0.518	-	-	-	-	-
Model 4	Case 1		5173	0.053	-	-	-	-	69.853
Model 11	Case 2		4045	0.196	-	-	-	-	266.921
Model 11	Case 3		10,766	0.065	-	-	-	-	0.259
Model 5	Case 1		5332	0.006	0.640	0.067	-	-	-
Model 12	Case 2		10,082	0.306	0.071	0.020	-	-	-
Model 12	Case 3		12,337	0.061	0.060	0.746	-	-	-
Model 6	Case 1		7457	-	0.552	0.021	0.038	-	-
Model 13	Case 2		8304	-	0.006	0.432	0.008	-	-
Model 13	Case 3		12,752	-	0.345	0.120	0.822	-	-
Model 7	Case 1		4942	-	0.725	0.044	0.018	1.00	-
Model 14	Case 2		11,233	-	0.009	0.201	0.004	0.00	-
Model 14	Case 3		12,653	-	0.093	0.430	0.104	0.00	-

**Table 7:** Parameter Estimates for Equinox.

Models	Prediction Classes	Actual Bugs	Parameter Estimates						
			*y*	*g*	*c*	*q*	*r*	*ψ*	*β*
Model 1	Case 1	8066	11,515	0.009	-	-	-	-	-
Model 8	Case 2		9567	0.002	-	-	-	-	-
Model 8	Case 3		12,531	0.076	-	-	-	-	-
Model 2	Case 1		8121	0.038	-	-	-	-	-
Model 9	Case 2		10,015	0.005	-	-	-	-	-
Model 9	Case 3		9360	0.259	-	-	-	-	-
Model 3	Case 1		7749	0.062	-	-	-	-	-
Model 10	Case 2		8588	0.009	-	-	-	-	-
Model 10	Case 3		8271	0.459	-	-	-	-	-
Model 4	Case 1		7782	0.048	-	-	-	-	6.259
Model 11	Case 2		14,583	0.002	-	-	-	-	1.743
Model 11	Case 3		15,143	0.094	-	-	-	-	0.961
Model 5	Case 1		7931	-	0.435	0.107	0.019	-	-
Model 12	Case 2		17,575	-	0.649	0.002	0.000	-	-
Model 12	Case 3		20,563	-	0.190	0.218	0.164	-	-
Model 6	Case 1		8207	-	0.232	0.677	0.031	-	-
Model 13	Case 2		17,900	-	0.041	0.025	0.040	-	-
Model 13	Case 3		19,612	-	0.370	0.108	0.809	-	-
Model 7	Case 1		7633	-	0.731	0.060	0.037	0.991	-
Model 14	Case 2		26,896	-	0.031	0.020	0.018	0.003	-
Model 14	Case 3		17,406	-	0.144	0.415	0.103	0.007	-

**Table 8:** Parameter Estimates for Orion.

Models	Prediction Classes	Actual Bugs	Parameter Estimates						
			*y*	*g*	*c*	*q*	*r*	*ψ*	*β*
Model 1	Case 1	7425	10,502	0.016	-	-	-	-	-
Model 8	Case 2		50,546	0.000	-	-	-	-	-
Model 8	Case 3		70,489	0.009	-	-	-	-	-
Model 2	Case 1		7573	0.058	-	-	-	-	-
Model 9	Case 2		9382	0.991	-	-	-	-	-
Model 9	Case 3		9382	0.006	-	-	-	-	-
Model 3	Case 1		7082	0.096	-	-	-	-	-
Model 10	Case 2		7813	0.011	-	-	-	-	-
Model 10	Case 3		8382	0.377	-	-	-	-	-
Model 4	Case 1		10,502	0.016	-	-	-	-	0.000
Model 11	Case 2		6043	0.037	-	-	-	-	1936.012
Model 11	Case 3		14,352	0.134	-	-	-	-	3.100
Model 5	Case 1		11,633	-	0.251	0.050	0.515	-	-
Model 12	Case 2		29,024	-	0.036	0.017	0.019	-	-
Model 12	Case 3		17,483	-	0.253	0.388	0.068	-	-
Model 6	Case 1		12,138	-	0.170	0.068	0.667	-	-
Model 13	Case 2		29,752	-	0.023	0.026	0.030	-	-
Model 13	Case 3		19,846	-	0.210	0.181	0.363	-	-
Model 7	Case 1		10,181	-	0.022	0.687	0.023	0.00	-
Model 14	Case 2		25,198	-	0.040	0.018	0.019	0.01	-
Model 14	Case 3		21,858	-	0.201	0.233	0.123	0.38	-

**Table 9:** Parameter Estimates for Platform.

Models	Prediction Classes	Actual Bugs	Parameter Estimates						
			*y*	*g*	*c*	*q*	*r*	*ψ*	*β*
Model 1	Case 1	39,671	38,839	0.014	-	-	-	-	-
Model 8	Case 2		77,829	0.000	-	-	-	-	-
Model 8	Case 3		52,937	0.083	-	-	-	-	-
Model 2	Case 1		34,392	0.037	-	-	-	-	-
Model 9	Case 2		49,091	0.001	-	-	-	-	-
Model 9	Case 3		40,539	0.277	-	-	-	-	-
Model 3	Case 1		33,245	0.061	-	-	-	-	-
Model 10	Case 2		40,691	0.002	-	-	-	-	-
Model 10	Case 3		36,771	0.475	-	-	-	-	-
Model 4	Case 1		38,839	0.014	-	-	-	-	0.000
Model 11	Case 2		65,121	0.001	-	-	-	-	2.239
Model 11	Case 3		66,742	0.064	-	-	-	-	0.076
Model 5	Case 1		38,193	-	0.474	0.031	0.233	-	-
Model 12	Case 2		145,329	-	0.024	0.005	0.007	-	-
Model 12	Case 3		66,742	-	0.076	0.164	0.064	-	-
Model 6	Case 1		38,381	-	0.135	0.106	0.219	-	-
Model 13	Case 2		118,171	-	0.012	0.012	0.009	-	-
Model 13	Case 3		89,932	-	0.358	0.104	1.000	-	-
Model 7	Case 1		37,297	-	0.268	0.760	0.017	0.88	-
Model 14	Case 2		98,807	-	0.008	0.024	0.003	0.00	-
Model 14	Case 3		70,590	-	0.087	0.549	0.104	0.00	-

**Table 10:** Performance Measures for BIRT.

Models	Prediction Classes	Performance Measures				
		*R* ^2^	Bias	MSE	Variation	RMSPE
Model 1	Case 1	0.954	−163.495	1,194,892.241	1084.230	1096.488
Model 8	Case 2	0.982	−17.696	453,766.553	675.517	675.749
Model 8	Case 3	**0.985**	−98.599	394,474.665	622.244	630.008
Model 2	Case 1	0.982	−7.395	453,323.773	675.380	675.420
Model 9	Case 2	**0.994**	69.679	146,078.571	376.984	383.370
Model 9	Case 3	**0.994**	74.541	150,867.384	382.401	389.599
Model 3	Case 1	0.941	99.646	541,276.388	731.239	737.997
Model 10	Case 2	**0.985**	122.257	375,407.367	602.281	614.564
Model 10	Case 3	**0.985**	127.544	381,748.506	606.460	619.727
Model 4	Case 1	0.986	1.244	351,146.125	594.447	594.448
Model 11	Case 2	**1.000**	23.738	38,455.058	195.273	196.710
Model 11	Case 3	0.999	42.930	12,988.803	105.907	114.277
Model 5	Case 1	0.984	12.355	405,231.593	638.469	638.589
Model 12	Case 2	0.985	4.661	390,022.091	626.473	626.491
Model 12	Case 3	**0.998**	12.001	57,487.729	240.222	240.522
Model 6	Case 1	0.984	23.937	415,426.803	646.127	646.570
Model 13	Case 2	**0.997**	96.403	83,831.124	273.878	290.350
Model 13	Case 3	0.990	246.435	266,484.564	455.035	517.481
Model 7	Case 1	0.985	2.000	390,062.997	626.520	626.524
Model 14	Case 2	0.996	−163.103	105,145.596	281.141	325.027
Model 14	Case 3	**0.999**	51.892	33,526.995	176.151	183.636

**Table 11:** Performance Measures for CDT.

Models	Prediction Classes	Performance Measures				
		*R* ^2^	Bias	MSE	Variation	RMSPE
Model 1	Case 1	0.936	−307.488	1,164,072.526	1034.178	1078.922
Model 8	Case 2	0.996	0.905	65,518.687	255.965	255.966
Model 8	Case 3	**1.000**	6.020	6334.770	79.363	79.591
Model 2	Case 1	**0.990**	−52.882	189,299.515	431.860	435.086
Model 9	Case 2	**0.990**	131.072	175,543.721	397.950	418.979
Model 9	Case 3	0.985	169.365	278,993.022	500.309	528.198
Model 3	Case 1	**0.993**	30.106	134,173.962	365.058	366.298
Model 10	Case 2	0.974	229.204	478,157.939	652.398	691.490
Model 10	Case 3	0.963	282.772	674,920.035	771.336	821.535
Model 4	Case 1	0.994	−18.394	117,906.308	342.882	343.375
Model 11	Case 2	**1.000**	46.601	8678.597	80.665	93.159
Model 11	Case 3	**1.000**	7.838	6412.712	79.695	80.079
Model 5	Case 1	0.993	−23.043	136,263.595	368.419	369.139
Model 12	Case 2	0.999	40.189	22,924.823	145.978	151.409
Model 12	Case 3	**1.000**	4.323	6046.042	77.636	77.756
Model 6	Case 1	0.993	25.781	127,341.016	355.916	356.849
Model 13	Case 2	**0.999**	26.686	30,079.843	171.370	173.435
Model 13	Case 3	**0.999**	63.130	24,504.164	143.244	156.538
Model 7	Case 1	0.988	−46.152	218,158.724	464.789	467.075
Model 14	Case 2	**1.000**	−0.397	1040.268	32.251	32.253
Model 14	Case 3	**1.000**	5.465	6319.237	79.306	79.494

**Table 12:** Performance Measures for Community.

Models	Prediction Classes	Performance Measures				
		*R* ^2^	Bias	MSE	Variation	RMSPE
Model 1	Case 1	0.870	−377.826	2,651,431.333	1583.881	1628.322
Model 8	Case 2	0.985	38.621	280,844.882	528.539	529.948
Model 8	Case 3	**0.990**	−110.623	212,211.498	447.185	460.664
Model 2	Case 1	**0.997**	46.663	53,829.332	227.270	232.011
Model 9	Case 2	0.988	176.111	248,543.613	466.399	498.541
Model 9	Case 3	0.986	189.262	282,806.236	496.977	531.795
Model 3	Case 1	**0.990**	141.903	213,061.200	439.232	461.586
Model 10	Case 2	0.968	287.249	645,863.042	750.567	803.656
Model 10	Case 3	0.967	141.903	213,061.200	439.232	461.586
Model 4	Case 1	0.998	−39.074	36,042.701	185.785	189.849
Model 11	Case 2	**1.000**	24.461	5645.190	71.041	75.134
Model 11	Case 3	**1.000**	24.311	7576.764	83.581	87.045
Model 5	Case 1	0.979	−49.445	426,340.972	651.073	652.948
Model 12	Case 2	0.998	58.820	40,477.491	192.400	201.190
Model 12	Case 3	**0.999**	12.254	11,536.524	106.707	107.408
Model 6	Case 1	0.990	22.104	194,491.013	440.457	441.011
Model 13	Case 2	**0.997**	43.838	63,797.015	248.747	252.581
Model 13	Case 3	0.992	164.222	164,655.758	371.062	405.778
Model 7	Case 1	0.959	260.299	838,248.268	877.777	915.559
Model 14	Case 2	**0.999**	23.633	15,895.979	123.845	126.079
Model 14	Case 3	**0.999**	24.528	10,470.253	99.341	102.324

**Table 13:** Performance Measures for EclipseLink.

Models	Prediction Classes	Performance Measures				
		*R* ^2^	Bias	MSE	Variation	RMSPE
Model 1	Case 1	0.890	−68.855	678,238.979	820.669	823.553
Model 8	Case 2	0.948	−21.774	323,428.341	568.291	568.708
Model 8	Case 3	**0.987**	10.891	79,180.202	281.179	281.390
Model 2	Case 1	0.934	137.527	407,579.417	623.431	638.419
Model 9	Case 2	**0.988**	108.322	123,830.963	334.809	351.896
Model 9	Case 3	0.985	94.631	95,383.522	293.987	308.842
Model 3	Case 1	0.898	205.498	634,357.651	769.499	796.466
Model 10	Case 2	0.958	165.170	259,656.410	482.053	509.565
Model 10	Case 3	**0.961**	153.624	238,577.579	463.656	488.444
Model 4	Case 1	0.969	54.919	192,514.466	435.314	438.765
Model 11	Case 2	0.997	27.387	20,699.431	141.242	143.873
Model 11	Case 3	**1.000**	8.512	2201.280	46.139	46.918
Model 5	Case 1	0.929	152.657	441,328.862	646.548	664.326
Model 12	Case 2	0.943	−95.121	353,611.586	586.995	594.652
Model 12	Case 3	**0.992**	54.938	49,197.141	214.893	221.804
Model 6	Case 1	0.950	1.184	307,477.827	554.506	554.507
Model 13	Case 2	0.955	165.150	280,454.177	503.170	529.579
Model 13	Case 3	**0.993**	77.748	44,252.107	195.467	210.362
Model 7	Case 1	0.940	91.341	373,154.792	603.996	610.864
Model 14	Case 2	0.957	155.151	263,713.386	489.532	513.530
Model 14	Case 3	**0.996**	20.478	23,867.677	153.128	154.492

**Table 14:** Performance Measures for EMF.

Models	Prediction Classes	Performance Measures				
		*R* ^2^	Bias	MSE	Variation	RMSPE
Model 1	Case 1	0.867	−194.454	566,339.067	726.998	752.555
Model 8	Case 2	0.992	−5.880	34,692.570	186.167	186.259
Model 8	Case 3	**0.998**	9.949	9583.560	97.389	97.896
Model 2	Case 1	0.953	−95.060	199,671.668	436.618	446.846
Model 9	Case 2	**0.999**	25.565	4533.714	62.291	67.333
Model 9	Case 3	0.990	38.686	41,304.357	199.519	203.235
Model 3	Case 1	0.971	−69.054	124,805.672	346.464	353.278
Model 10	Case 2	**0.997**	32.121	13,362.272	111.043	115.595
Model 10	Case 3	0.982	47.216	74,767.394	269.329	273.436
Model 4	Case 1	0.980	−35.506	86,137.501	291.336	293.492
Model 11	Case 2	0.790	5.153	895,970.753	946.543	946.557
Model 11	Case 3	**0.998**	14.328	10,192.590	99.936	100.958
Model 5	Case 1	0.945	−36.387	234,715.476	483.106	484.474
Model 12	Case 2	0.987	−57.478	55,974.800	229.502	236.590
Model 12	Case 3	**0.998**	12.348	9539.927	96.889	97.673
Model 6	Case 1	0.966	−63.531	143,888.622	373.968	379.327
Model 13	Case 2	**0.999**	27.787	4036.419	57.134	63.533
Model 13	Case 3	0.997	26.620	13,777.412	114.319	117.377
Model 7	Case 1	0.931	−3.437	292,928.848	541.218	541.229
Model 14	Case 2	**1.000**	6.864	316.260	16.406	17.784
Model 14	Case 3	0.998	11.915	9511.022	96.794	97.524

**Table 15:** Performance Measures for Equinox.

Models	Prediction Classes	Performance Measures				
		*R* ^2^	Bias	MSE	Variation	RMSPE
Model 1	Case 1	0.962	−40.095	242,895.945	491.211	492.845
Model 8	Case 2	0.956	−56.757	278,566.818	524.734	527.794
Model 8	Case 3	**0.989**	−6.788	68,392.382	261.431	261.519
Model 2	Case 1	**0.992**	13.917	51,576.341	226.677	227.104
Model 9	Case 2	0.989	87.786	72,045.729	253.652	268.413
Model 9	Case 3	0.987	91.327	81,140.316	269.814	284.851
Model 3	Case 1	**0.991**	63.199	59,405.042	235.395	243.731
Model 10	Case 2	0.973	145.920	171,480.372	387.541	414.102
Model 10	Case 3	0.970	152.727	193,297.437	412.277	439.656
Model 4	Case 1	0.993	9.389	45,949.640	214.153	214.359
Model 11	Case 2	**1.000**	14.159	1736.049	39.186	41.666
Model 11	Case 3	**1.000**	6.741	1489.955	38.007	38.600
Model 5	Case 1	0.993	22.176	43,161.357	206.566	207.753
Model 12	Case 2	0.993	−68.241	42,137.573	193.599	205.274
Model 12	Case 3	**1.000**	4.367	1152.694	33.669	33.951
Model 6	Case 1	0.994	44.660	37,420.240	188.217	193.443
Model 13	Case 2	**0.997**	11.406	18,191.483	134.393	134.876
Model 13	Case 3	**0.997**	45.049	17,913.479	126.032	133.841
Model 7	Case 1	0.991	82.876	56,042.397	221.752	236.733
Model 14	Case 2	**1.000**	12.361	4062.648	62.529	63.739
Model 14	Case 3	**1.000**	3.191	1167.919	34.025	34.175

**Table 16:** Performance Measures for Orion.

Models	Prediction Classes	Performance Measures				
		*R* ^2^	Bias	MSE	Variation	RMSPE
Model 1	Case 1	0.995	18.588	23,679.888	152.756	153.883
Model 8	Case 2	**0.998**	−22.362	7555.096	83.994	86.920
Model 8	Case 3	0.993	−37.593	32,104.300	175.189	179.177
Model 2	Case 1	**0.996**	116.875	151,851.918	371.742	389.682
Model 9	Case 2	0.991	41.627	39,464.244	194.246	198.656
Model 9	Case 3	0.991	44.431	35,004.897	181.744	187.096
Model 3	Case 1	0.932	181.410	309,045.757	525.487	555.919
Model 10	Case 2	0.973	81.913	121,771.764	339.208	348.958
Model 10	Case 3	**0.977**	83.590	102,003.389	308.247	319.380
Model 4	Case 1	0.995	181.410	309,045.757	525.487	555.919
Model 11	Case 2	0.769	300.134	1,042,674.949	976.009	1021.115
Model 11	Case 3	**1.000**	4.173	2098.407	45.618	45.808
Model 5	Case 1	0.984	−2.093	17,985.360	134.093	134.110
Model 12	Case 2	**1.000**	−0.863	1212.484	34.810	34.821
Model 12	Case 3	0.999	6.399	2300.080	47.530	47.959
Model 6	Case 1	0.996	2.455	17,943.138	133.929	133.952
Model 13	Case 2	**1.000**	8.771	1822.765	41.783	42.694
Model 13	Case 3	0.995	36.389	22,420.814	145.247	149.736
Model 7	Case 1	0.995	19.784	23,315.361	151.407	152.694
Model 14	Case 2	**1.000**	8.032	1946.990	43.387	44.125
Model 14	Case 3	0.999	6.208	2396.167	48.555	48.951

**Table 17:** Performance Measures for Platform.

Models	Prediction Classes	Performance Measures				
		*R* ^2^	Bias	MSE	Variation	RMSPE
Model1	Case 1	0.988	−60.812	1,253,249.581	1117.833	1119.486
Model 8	Case 2	0.986	−225.334	1,406,893.822	1164.525	1186.126
Model 8	Case 3	**0.992**	390.633	760,803.806	779.878	872.241
Model 2	Case 1	0.968	269.670	3,202,764.241	1769.193	1789.627
Model 9	Case 2	**0.988**	251.961	1,238,555.994	1084.007	1112.904
Model 9	Case 3	0.981	349.006	1,960,595.755	1356.020	1400.213
Model 3	Case 1	0.941	454.483	6,030,048.031	2413.192	2455.616
Model 10	Case 2	**0.968**	439.203	3,195,122.116	1732.692	1787.490
Model 10	Case 3	0.954	574.790	4,688,401.115	2087.587	2165.272
Model 4	Case 1	0.988	−60.812	1,253,249.581	1117.833	1119.486
Model 11	Case 2	0.999	61.744	68,204.084	253.755	261.159
Model 11	Case 3	**1.000**	41.716	17,430.761	125.262	132.026
Model 5	Case 1	0.988	−8.745	1,195,080.065	1093.162	1093.197
Model 12	Case 2	0.995	−14.352	124,215.189	352.149	352.442
Model 12	Case 3	**0.998**	−454.100	465,100.649	508.816	681.983
Model 6	Case 1	0.988	5.076	1,188,051.280	1089.966	1089.978
Model 13	Case 2	0.998	108.426	210,247.736	445.524	458.528
Model 13	Case 3	**0.999**	80.470	137,436.012	361.885	370.724
Model 7	Case 1	0.989	114.197	1,134,247.913	1058.871	1065.011
Model 14	Case 2	0.998	51.394	216,738.414	462.706	465.552
Model 14	Case 3	**1.000**	20.934	13,982.125	116.378	118.246

## Abbreviations

**Acronyms**	
OSS	Open Source Software
NHPP	Non Homogenous Poisson Process
SPSS	Statistical Package for Social Sciences
SRGM	Software Reliability Growth Model
**Notations**	
*t*	Time
*y*/*p*	Potential number of bugs lying dormant in the software that can be fixed over a long run
*y*_1_/*p*_1_	Number of independent bugs
*y*_2_/*p*_2_	Number of dependent bugs
*x*(*t*)	Mean value function of bug detection/fixed up to time *t*
*x*_1_(*t*)	Mean value function of the expected number of independent bugs
*x*_2_(*t*)	Mean value function of the expected number of dependent bugs
*g*/*k*	Rate of bug detection/fixed
*r*/*l*	Rate of bug detection/fixed of independent bugs
*c*/*d*	Rate of bug detection/fixed of dependent bugs
*θ*(*t*)/*θ*(*H*(*t*))	Delay–effect factor i.e., debugging time lag
*ψ*	Inflection factor
*q*	Proportion of the independent bugs
*β*	Constant and >0
*H*(*t*) or H(*t*)	The value of summary/comment entropy at time *t* be consistent writing *H* or H
*x*(H(*t*))	Bugs removed by cumulative entropy value H(t)
*δ*	Constant and >0
